# Definition by FSH, AMH and embryo numbers of good-, intermediate- and poor-prognosis patients suggests previously unknown IVF outcome-determining factor associated with AMH

**DOI:** 10.1186/s12967-016-0924-7

**Published:** 2016-06-10

**Authors:** Norbert Gleicher, Vitaly A. Kushnir, Aritro Sen, Sarah K. Darmon, Andrea Weghofer, Yan-Guang Wu, Qi Wang, Lin Zhang, David F. Albertini, David H. Barad

**Affiliations:** The Center for Human Reproduction, 21 East 69th Street, New York, NY 10021 USA; The Foundation for Reproductive Medicine, New York, NY USA; Stem Cell Biology and Molecular Embryology Laboratory, The Rockefeller University, New York, NY USA; Department of Obstetrics and Gynecology, Wake Forest University, Winston Salem, NC USA; Division of Medical Endocrinology and Metabolism, Department of Medicine, Rochester University School of Medicine and Dentistry, Rochester, NY USA; Vienna University School of Medicine, Vienna, 1090 Austria; Department of Molecular and Integrative Physiology, The University of Kansas Medical Center, Kansas City, KS USA; Department of Obstetrics and Gynecology, Albert Einstein College of Medicine, Bronx, NY USA

**Keywords:** In vitro fertilization (IVF), Prognosis, Live birth rates, Prediction models, Anti-Müllerian hormone (AMH), Follicle stimulating hormone (FSH), Embryo number

## Abstract

**Background:**

Though outcome models have been proposed previously, it is unknown whether cutoffs in clinical pregnancy and live birth rates at all ages are able to classify in vitro fertilization (IVF) patients into good-, intermediate- and poor prognosis.

**Methods:**

We here in 3 infertile patient cohorts, involving 1247, 1514 and 632 women, built logistic regression models based on 3 functional ovarian reserve (FOR) parameters, including (1) number of good quality embryos, (2) follicle stimulating hormone (FSH, mIU/mL) and (3) anti-Müllerian hormone (AMH, ng/mL), determining whether clinical pregnancy and live birth rates can discriminate between good, intermediate and poor prognosis patients.

**Results:**

All models, indeed, allowed at all ages for separation by prognosis, though cut offs changed with age. In the embryo model, increasing embryo production resulted in linear improvement of IVF outcomes despite transfer of similar embryo numbers; in the FSH model outcomes and FSH levels related inversely, while the association of AMH followed a bell-shaped polynomial pattern, demonstrating “best” outcomes at mid-ranges. All 3 models demonstrated increasingly poor outcomes with advancing ages, though “best” AMH even above age 43 was still associated with unexpectedly good pregnancy and delivery outcomes. Excessively high AMH, in contrast, was at all ages associated with spiking miscarriage rates.

**Conclusions:**

At varying peripheral serum concentrations, AMH, thus, demonstrates hithero unknown and contradictory effects on IVF outcomes, deserving at different concentrations investigation as a potential therapeutic agent, with pregnancy-supporting and pregnancy-interrupting properties.

**Electronic supplementary material:**

The online version of this article (doi:10.1186/s12967-016-0924-7) contains supplementary material, which is available to authorized users.

## At a glance commentary, Gleicher N et al

### Background

Prediction of IVF outcomes in patients at different ages has been a longstanding goal in reproductive medicine. Here we demonstrate that, based on embryo numbers produced (retroactive prediction paradigm), FSH and AMH levels (both prospective prediction paradigms), different models are predictive of good, intermediate and poor IVF prognoses at all ages.

### Translational significance

This is the first study to demonstrate prospective age-specific IVF outcome predictions based on FSH and AMH levels, and a retrospective prediction model based on embryo numbers.

In an unexpected translational finding, AMH was found associated with contradictory IVF outcomes at "best" (unexpectedly high pregnancy and delivery rates) and excessively high peripheral serum levels (spiking spontaneous miscarriages).

If confirmed, these observation suggest the potential clinical use of AMH as a fertility enhancing pharmacological agent at "best" levels and as a potential abortefaciant at excessively high levels.

## Background

How to establish outcome prognoses for infertile women entering in vitro fertilization (IVF) cycles is not well defined [1]. If accomplishable at all ages, the ability to predict prognoses would, therefore, be clinically very valuable. Better definitions of patient populations at IVF centers would also improve internal as well as external quality controls. In the US such external controls are mandated by an act of Congress [2], and currently not satisfactory [3]. Finally, treatments offer different levels of efficacy in good-, intermediate- and poor-prognosis patients [4]. Better definition of “disease” severity, therefore, should improve individualization of IVF treatments and, thereby, improve outcomes.

Prognostication of IVF outcomes has been a longstanding goal [5]. With key component female age [6, 7, 9], a variety of models have been published [6–11] as declining clinical pregnancy and live birth rates with advancing female age well demonstrate [12]. te Velde et al. [10] therefore, were correct in noting that, when building prediction models for IVF, changes in outcomes have to be considered with advancing female age.

Age is, however, not the only important predictor of IVF outcomes. Functional ovarian reserve (FOR), a term reflecting the growing follicle pool, and, therefore, oocyte and embryo numbers, is also closely associated with IVF outcomes. Abnormally low FOR (LFOR) is defined by abnormally increased age-specific follicle stimulating hormone (FSH) [13] and/or decreased age-specific anti-Müllerian hormone (AMH) [14], both reflecting declining egg and embryo numbers and, therefore, deteriorating pregnancy and live birth chances [15].

In women with premature ovarian aging (POA), also called occult primary ovarian insufficiency (oPOI) [16], normal statistical associations between age and FOR are disturbed. POA/oPOI patients prematurely demonstrate LFOR. They represent approximately 10 % of females, independent of race and ethnicity, and at IVF centers can exceed half of all patients [17]. In POA patients FOR-based rather than age-based prediction models in IVF may, therefore, be preferable.

We here present three different models, involving age and FOR, which based on clinical pregnancy as well as live birth chances allow definitions of patients into good-, intermediate and poor IVF prognosis categories. The here presented study yielded in addition unexpected results, which suggest previously unrecognized physiologic effects of AMH on IVF outcomes.

## Methods

### Patient populations

This study involves three partially overlapping patient cohorts: Cohort I, 1247 consecutive fresh IVF cycles during 2009–2013, including egg donor, however excluding elective single embryo transfer (eSET) and mild stimulation cycles, was used to investigate associations of good quality embryo numbers (between 1 and 15) with clinical pregnancy and live birth rates at different ages (<36, 36–38, 39–40, 41–42 and ≥43 years). Patients <36 are presented as a single age category because ages <30, 31–32, 33–34 and 35–36 produced basically identical outcomes (see Additional file 1: Appendix Figure S1).

Cohort II, 1514 consecutive fresh autologous non-donor IVF cycles, excluding eSET and mild stimulation cycles, was stratified for age used to establish associations of highest FSH levels (2.5–40.0 mIU/mL) with clinical pregnancy and live birth rates.

Cohort III, 632 fresh autologous non-donor cycles between 2011 and 2014, excluding eSET and mild stimulation cycles, was used to assess associations of lowest AMH levels (≤0.5–10.0 ng/mL) with clinical pregnancy and live birth rates, stratified for age. Only AMH measurements by the Beckman Generation 2 AMH assay were included as neither manufacturers of earlier AMH assays nor our own statisticians able to generate conversion tables.

All patient data, representing consecutive IVF cycles, were extracted from our center’s anonymized electronic research data base unless meeting the exclusion criteria noted above. Table 1 summarizes patient and IVF cycle characteristics for all three patient cohorts.

**Table 1:** Patient characteristics of patient Cohort I, II and III

	Cohort I	Cohort II	Cohort III
Cycles (n)	1247	1514	632
Embryos (n)	4.9 ± 4.3	4.0 ± 3.4	4.1 ± 3.5
Age (years)	37.8 ± 6.7	39.5 ± 5.0	39.5 ± 4.9
FSH (mIU/mL)	17.3 ± 19.1	15.4 ± 14.8	15.8 ± 16.2
AMH (ng/mL)	1.1 ± 1.9	1.1 ± 1.9	1.1 ± 1.9
Pregnancies			
n	346	246	106
%	27.8	16.2	16.8
Live births			
n	264	178	73
%	21.2	11.8	11.6
Miscarriages			
n	82	68	33
%	23.7	27.6	31.3

### FSH and AMH values

FSH values were tested in house by commercial assay. Though commercial AMH assays are similar at mid-range (variations between assays are usually only seen at very low and very high levels), here reported values should not automatically be applied to other AMH assays since earlier generation AMH assays differ from the in this study utilized assay. We later demonstrate that, indeed, mid-range values matter most in here reported AMH model.

Blood draws occurred at initial presentation, with IVF cycle starts on average initiating 8 weeks later.

### IVF cycle protocols

Cycle stimulation protocols at our center are limited, while choice of gonadotropin manufacturer is left up to patients and their insurance coverage. Oocyte donors receive a long agonist protocol (150–300 IU of gonadotropins daily), usually given as human menopausal gonadotropin (hMG). Since most of our center’s patients present with LFOR, a majority receive short microdose agonist protocols, with FSH (300–450 IU) and hMG (150 IU). Patients with normal FOR, if under age 38, receive similar stimulation to egg donors. Patients with LFOR are pretreated with dehydroepiandrosterone (DHEA) to raise testosterone levels to above 28 ng/mL (1 nmol/L) before IVF cycle start [18], and also receive CoQ10 supplementation [19].

Up to age 38, our center transfers in fresh cycles only 1–2 embryos; between ages 38–42, 3 embryos and above age 42, 3 to maximally 5 embryos.

### Embryo assessment

After assessment and grading, our center routinely transfers embryos on day-3 (cleavage stage) [20]. Only 4—8-cell embryos of at least grade 3 are transferred or cryopreserved and, therefore, considered good quality.

### Statistics

FOR parameters and categorical age were used to model the probability of clinical pregnancy, live birth or pregnancy loss using logistic regression. For models with AMH, AMH [2] was also included, and a statistically significant predictor of all outcomes. A P value of <0.05 was considered statistically significant. All statistical analyses were performed by the center’s senior statistican (S.K.D.), using SAS version 9.4 software.

### Ethics, consent and permissions

Patients whose data are preserved in our center’s anonymized electronic database sign at presentation an informed consent that allows use of their medical records for research, as long as their anonymity is preserved and their medical records remains confidential. Both conditions are met when data is extracted from the electronic database. Such projects are, therefore, approved by the center’s IRB (IRB of The Center for Human Reproduction, Chairman, Neil Rosenberg, MD) as expedited applications. This here presented study was, thus, approved under IRB application number ER0330215/01.

## Results and discussion

### Effects of embryo numbers

Table 2 summarizes cycle characteristics for Cohort I. As expected, good quality embryos, pregnancy and live birth rates declined with advancing age, while miscarriage rates increased. In age-specific categories, miscarriages can be defined in all figures as the differences between age-specific clinical pregnancy and live birth rates.

**Table 2:** Cycle outcome characteristics for Cohorts I, II and III in age categories

Ages (years)	<36	36–38	39–40	41–42	≥43
COHORT I (n = 1247)					
Cycles (n)	432	174	162	183	296
Embryos (n)	7.5 ± 5.1	3.7 ± 2.9	3.7 ± 2.9	3.3 ± 2.4	3.1 ± 2.2
Pregnancies					
n	203	49	40	32	22
%	47.0	28.2	24.7	17.5	7.4
Live births					
n	173	34	29	18	10
%	40.0	19.5	17.9	9.8	3.4
Miscarriages					
n	30	15	11	14	12
%	14.8	30.6	27.8	43.8	54.6
COHORT II (n = 1514)					
Cycles (n)	303	234	239	259	479
FSH (mIU/mL)	11.8 ± 12.7	15.0 ± 14.1	15.5 ± 13.2	14.7 ± 14.2	18.0 ± 16.9
Pregnancies					
n	101	50	40	32	23
%	33.3	21.4	16.7	12.4	4.8
Live births					
n	86	35	29	18	10
%	28.4	15.0	12.1	6.9	2.1
Miscarriages					
n	15	15	11	14	13
%	14.9	30.0	27.5	43.8	56.5
COHORT III (n = 632)					
Cycles (n)	127	87	103	109	206
AMH (ng/mL)	2.3 ± 3.3	1.1 ± 1.9	0.7 ± 1.0	0.6 ± 1.1	0.7 ± 0.7
Pregnancies					
n	45	16	16	14	15
%	35.4	18.4	15.5	12.8	7.3
Live births					
n	37	11	13	7	5
%	29.1	12.6	12.6	6.4	2.4
Miscarriages					
n	8	5	3	7	10
%	17.8	31.2	18.8	50.0	66.7

**Fig. 1:**
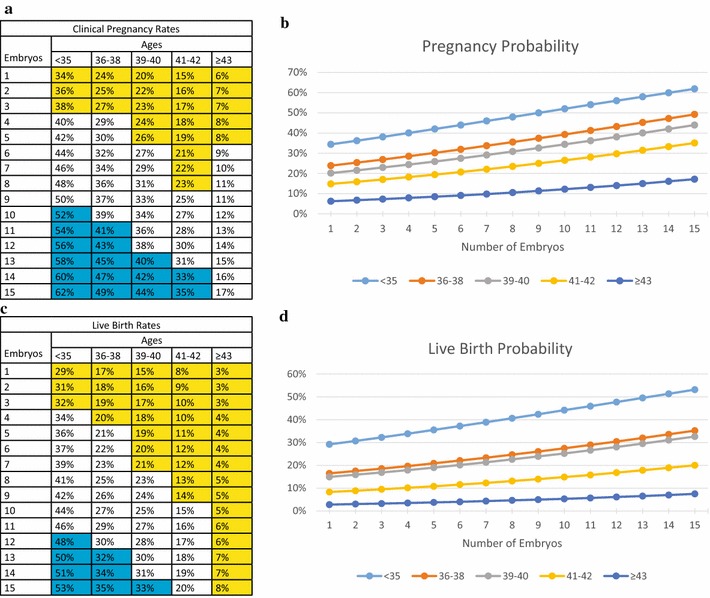
Age-specific model of pregnancies and live births based on good quality embryos produced per cycle. **a**, **b** reflect clinical pregnancy rates; **c**, **d** reflect live birth rates; In (**a**) and (**c**), *blue* background denotes good-prognosis, *white* denoted intermediate- and *yellow* poor-prognosis

How embryo numbers affected clinical pregnancy and live birth rates is shown in Fig. 1: Good-, intermediate- and poor-outcomes within each age group were defined at visually obvious break points in pregnancy and live birth rates. In all figures, fields were colored in yellow for poor prognosis, in blue for good prognosis and left uncolored for intermediate-prognosis.

As Fig. 1a, c demonstrate, at youngest age (<36 years) pregnancy and delivery rates were excellent almost independent of good quality embryo numbers. Even poor prognosis patients (defined by only 1–3 embryos) still achieved clinical pregnancy rates of 34–38 % and live birth rates of 29–32 %. Both rates steadily increased with increasing embryo production to a maximum of 62 and 53 %, respectively.

Because our center only rarely performs elective single embryo transfer (eSET) [21], and up to age 38 practically never transfers more than 2 embryos, this age category at most received 2-embryo transfers (2ETs). Yet, pregnancy and live birth rates increased almost linearly (Fig. 1b, d) with increasing embryo production.

The pregnancy loss (miscarriage) rate, defined as clinical pregnancies minus live births, however remained similar, whether a woman produced 1 or 15 embryos: Pregnancy loss <36, for example, occurred in 14.7 % of women with 1 embryo and in 14.5 % of women with 15 embryos.

Figure 1a and c also demonstrate that, despited uniformly good clinical pregnancy and live birth rates <36 years, separation of good-prognosis (≥51 % clinical pregnancy and ≥44 % live birth), intermediate prognosis (respectively 40–50 and 34–44 %) and poor prognosis patients (respectively ≤39 and ≤33 %) was still possible based on obvious break points in cycle outcomes.

From age 36, outcomes started declining, while pregnancy losses increased, at age 36–38 reaching 29.2 % for women who produced 1, and 28.6 % in those with 15 embryos. This traditional embryo quality parameter, thus, remained stable (Fig. 1a, c) and almost linear improvements of pregnancy and live birth rates with increasing embryo production was maintained into older ages (Fig. 1b, d). Indeed, improvements within age categories between 1 and 15 embryos grew with advancing age: Under age 36, clinical pregnancy chances increased by 82.4 % (from 34 to 62 %) but by 104.2 % (from 24 to 49 %) in age category 36–38. Concomittantly, live birth rates improved by 89.3 % (from 29 to 53 %) under age 36 and by 105.9 % (from 17 to 35 %) at ages 36–38. By age ≥43, clinical pregnancy rate for 1 embryo was 6 %, and for 15 embryos 17 %, a 183 % increase, while live births increased from 3 to 8 %, a 166.7 % increase (Fig. 1).

As Fig. 1a and c demonstrate, with persistently decreasing clinical pregnancy and live birth rates, women ≥43 years only with 7 or more embryos reached 10 % clinical pregnancy rates or higher, and even with up to 15 embryos remained in single digit range for live births. No woman in that age group, therefore, could be considered a good prognosis patient.

Increasingly, poor embryo quality with advancing female age was also reflected in increasing pregnancy loss, in women with 1 embryo reaching 50.0 % at age ≥43, and 52.9 % with 15 embryos. The embryo quality parameter of pregnancy loss, therefore, remained similar within age categories,—even at most advanced age; yet, clinical pregnancy and live birth rates within age categories improved with growing embryo numbers produced, and did so increasingly more pronounced as women grew older.

### Effects of FSH levels

Table 2 describes cycle numbers, peak FSH levels and clinical pregnancy as well as live birth rates at different female ages.

**Fig. 2:**
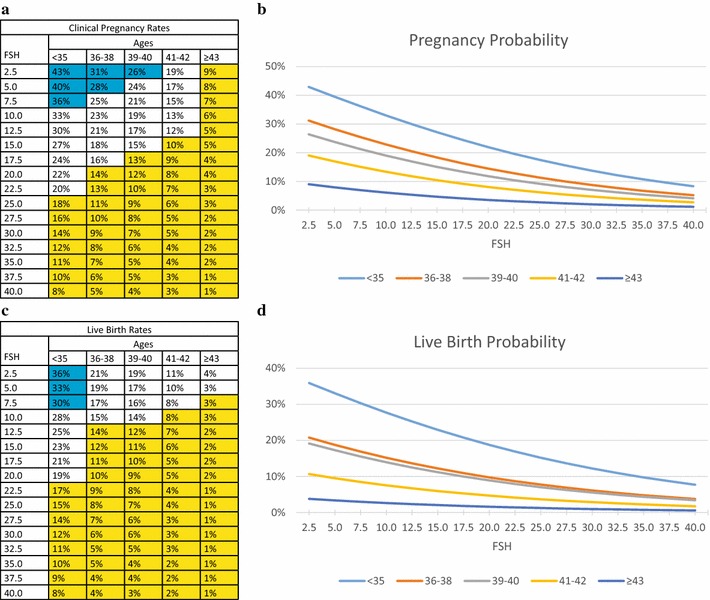
Age-specific model of pregnancies and live births based on FSH levels (in mIU/nL). **a**, **b** Reflect clinical pregnancy rates; **c**, **d** reflect live birth rates; In (**a**) and (**c**) *blue* background denotes good-prognosis, *white* background intermediate- and *yellow* background poor-prognosis

Figure 2 summarizes probabilities of clinical pregnancies (Fig. 2a, b) and live births (Fig. 2c, d) at FSH levels between 2.5 and 40.0 mIU/mL. Both at all ages declined with increasing FSH levels. Moreover, within each FSH category, both outcomes also declined with advancing age.

For women <36 years, FSH only up to 7.5 mIU/L denoted good prognosis (pregnancy 36–43 %; live birth 30–36 %). FSH levels mattered at all ages, with lower FSH levels, even with good-prognosis and within normal FSH levels offering better outcomes. Pregnancy after age 40, and live births even as early as age 36, failed to reach good-prognosis at even lowest FSH, suggesting that, at least in adversely selected patients, normal FSH levels may have to be reconsidered.

FSH changed in its clinical relevance with advancing female age: For example, an FSH of 22.5–25.0 mIU/mL; <36 years resulted in clinical pregnancy in 19 %; though at age 41–42, the same rate required an FSH of 2.5 mIU/mL (Fig. 2a); FSH of 32.5 mIU/mL <36 years, allowed live births in 11 %; but at 41–42, this live birth rate required an FSH of 2.5 mIU/mL (Fig. 2c).

Figure 2 also demonstrates that ≥43 years treatment futility, according to the American Society for Reproductive Medicine (ASRM) at ca. 1 % live birth rate [22], was reached at FSH 22.5 mIU/mL. Yet, up to 42 years, even up to FSH 40.0 mIU/mL futility was avoided.

### Effects of AMH levels

**Fig. 3:**
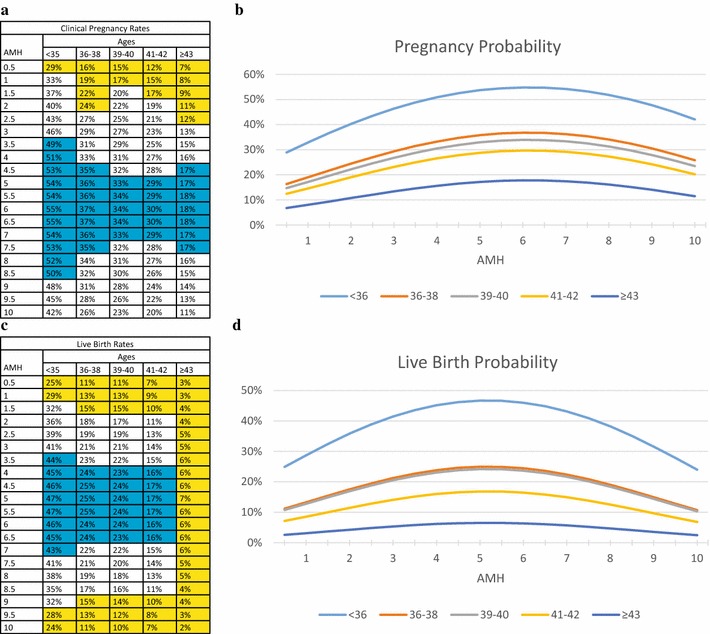
Age-specific model of pregnancies and deliveries based on AMH levels (in ng/ml). **a**, **b** Reflect clinical pregnancy rates; **c**, **d** reflect live birth rates; In (**a**) and (**c**) *blue* background denotes good prognosis, *white* average- and *yellow* poor-prognosis patients

Table 2 also introduces Cohort III, which was used to assess associations of a patient’s lowest AMH (between ≥0.5 and 10.0 ng/mL) with pregnancy (Fig. 3a, b) and live birth rates (Fig. 3c, d). In contrast to embryo and FSH models, pregnancy and live birth chances in association with AMH followed a bell-shaped curve, with best outcomes at midrange.

The very high pregnancy and live birth rates at “best” AMH levels were unexpected: Under age 36, AMH values between 3.5 ng/mL and 8.5 ng/mL offered best pregnancy chances (49–55 %, good-prognosis patients); 1.0–3.0, and 9.0–10.0 ng/mL offered intermediate-prognoses (pregnancy 33–46 %), and only AMH of ≤0.5 ng/mL denoted poor prognosis (with still respectable pregnancy rate of 29 %, Fig. 3a).

Live births behaved similarly (Fig. 3b): best live birth rates (43–47 %) were obtained at AMH 3.5–7.0 ng/mL; intermediate rates (32–41 %) at AMH of 1.5–3.0 and 7.5–9.0 ng/mL. Even poor prognosis at AMH of ≤1.0 ng/mL still was associated with 25–29 % live births.

Clinical pregnancy and live birth declined only mildly up to age 42, and an unexpectedly high 18 % pregnancy rate was still achieved ≥43. Live births reached a respectable 7 % (oldest conception at age 47). Pregnancies in single digits occurred only with AMH <1.5 ng/mL. With AMH ≥2.0 ng/mL, clinical pregnancy rates were between 10–18 %, though declined at very high AMH (Fig. 3a) after reaching peak pregnancy rates at AMH 5.5–6.5 ng/mL. This patient group, however, also experienced the highest pregnancy loss rate of any model.

Pregnancy loss at all ages remained similar for low and “best” AMH levels but significantly increased at highest AMH levels: <36 years, at AMH 0.5 ng/mL only 13.8 % miscarried and at “best” level of 5.5 ng/mL only 13.0 %; but at AMH of 10.0 ng/mL, rates spiked to 42.9 %. The same occurred ≥43, where pregnancy loss was 57.1 % with lowest AMH, 61.1 % at “best” AMH levels and spiked to 81.8 % at highest AMH, contradicting that AMH linearly reflects not only oocyte quantity but also quality (23).

### Statistical comments

Because all three here utilized statistical models highly correlate in representation of FOR in association with patient age, construction of combined statistical models was not feasable. In univariate models, FSH and AMH, independently, were not predictive of miscarriage. Embryo numbers, however, did reach significance in an univariate model (P = 0.008), though, as expected, significance was lost with adjustments for age, as embryo numbers in themselves are age-dependent.

### General discussion

This study was initiated to determine whether, even in relatively adversely selected infertile patient populations, definitions of good-, intermediate- and poor-prognosis can at different ages be reached based on clinical pregnancy and live birth rates. To the best of our knowledge, such an age-based association study has never before been performed, and certainly not in poor prognosis IVF patients. Here studied populations’ relatively poor prognoses are best demonstrated by their elevated FSH and abnormally low AMH levels (Table 1).

To be able to classify patients prospectively would be clinically useful for patients and physicians alike. To classify patient populations retrospectively, would allow for their better definition and, therefore, hypothetically for better outcome comparisons between IVF centers. To allow for such comparisons, national outcome reporting in the US is legislatively mandated by Congress [2], though the current system has recently been described as inadequate, and even misleading [3].

A final reason for this study was the recent recognition that some treatment effects vary between good-, intermediate- and poor-prognosis patients. Indeed, especially in poor prognosis patients, some widely utilized treatments may be outright harmful [4].

### Definition of patient prognosis

Since prospective definition of prognosis of IVF patients has been a longstanding goal, various models have been proposed [6–11]. None so far have, however, proven clinically effective [23, 24].

By assessing the impact of FOR on IVF outcome in three distinctively different models, this study, therefore, approached the issue differently: In a retrospective model, based on number of embryos produced in a given IVF cycle; and in two prospective models, utilizing FOR’s two most widely used laboratory surrogates, FSH and AMH.

This multifocal evaluation of FOR proved successful since, based on breakpoints in clinical pregnancy and live birth rates, it allowed in in each age category for differentiation between good-, intermediate- and poor-prognosis patients.

Defining parameters for individual prognosis categories, as expected [1] changed with advancing female age, and required increasing embryo numbers to maintain designations. With clinical pregnancy as final outcome, women ≥43 years no longer demonstrated what could be defined as good prognoses. With live births as final outcome, all women in that age category, indeed, demonstrated poor prognosis.

Utilizing peak FSH levels as FOR surrogate, similar associations became apparent (Fig. 2): pregnancy and live birth rates declined with increasing FSH and advancing age. Again, prognoses could be defined based on rather obvious cut offs in pregnancy and live birth rates. This model, however, already at young ages revealed a surprisingly narrow range of good-prognosis: in women <36 years, only FSH ≤ 7.5 mIU/mL, and at ages 36–37, only FSH ≤ 2.5 mIU/mL qualified as good-prognosis with reference pregnancy, while with end point live births, only age <36 qualified. Even intermediate-prognosis became rare after age 40, and required FSH levels <5.0 mIU/mL, while only poor-prognosis patients were left ≥43 years.

These data confirm the importance of utilization age-specific FSH levels in assessing infertile women [13].

More surprising observations were made in the AMH model: In contrast to the embryo and FSH models, it demonstrated a typical bell-shaped polynomial pattern. Worst IVF outcomes were observed at AMH extremes; “best” AMH was slightly above mid-point (Fig. 3). In pregnancy rates, this pattern carried over into the oldest patient group (Fig. 3a), though based on live births, no good prognosis patients were found ≥43 years (Fig. 3c).

Here reported outcomes are, of course, not automatically applicable to other IVF programs. They were the consequence of very specific practice patterns [18, 25]. Even assuming identical patient populations (in itself also a highly unlikely proposition), different clinical protocols at other centers will result in different pregnancy and live birth rates. To construct universally applicable models, this study will have to be repeated on a multicenter or even national basis, and further validated against results from IVF centers with varying patient populations and treatment protocols.

Different AMH assays utilized by IVF centers may also offer mildly varying results [26], though mid-range AMH, in this study demonstrated to be most important AMH range, demonstrates least discrepancies between currently in use AMH assays.

### Relevance of treatment protocols

Reliable prognostication of patients is of potential clinical importance: Treatments, which recently entered routine IVF, have shown varying effectiveness in different patient categories. For example, the concept of embryo selection in all of its applications appears beneficial only in good-prognosis patients. With intermediate-prognosis, embryo selection appears ineffective, while with poor prognosis it outright decreases pregnancy and live birth chances [4]. At the other extreme, treatments reported effective in adversely selected patients [18, 19, 25], may be ineffective in intermediate and good prognosis patients.

IVF protocols, therefore, have to evolve toward individualization of care, and a reproducible classification of patients, as here presented, would greatly contribute to standardization of individualized treatment options.

### Previously unknown AMH associations with IVF outcomes

Likely the clinically most consequential and translationally most important findings of this study relate to AMH levels: While in embryo and FSH models relationships were almost linear, clinical pregnancy and live birth chances in relation to AMH levels followed a bell-shaped curve, with maximal clinical pregnancy and live birth chances at midrange AMH, rather than highest or lowest levels.

Even into oldest age categories, this model at “best” AMH levels demonstrated unexpectedly high pregnancy rates. Live birth rates behaved similarly, and were remarkably high up to age 42. Beyond age 42, miscarrage rates, however, even at “best” AMH levels were extremely high. At “best” AMH levels, women ≥43, for example, reached an almost incredolous 18 % clinical pregnancy rate; but only a 7 % live birth rate, representing a 61.1 % clinical miscarriage rate. Though a 7 % live birth rate in this age category is still remarkable, the spike in observed pregnancy loss is even more stunning.

Though in embryo and FSH models pregnancy, independent of embryo numbers and FSH levels, loss rates remained the same within all age categories, in the AMH model miscarriage rates remained similar only at low (57.1 %) and “best” AMH levels (61.1 %); at highest AMH levels, they spiked to an incredible 81.8 %.

Combined, these AMH-associations suggest positive effects on clinical pregnancy and live birth rates up to “best” AMH levels but, because of increasing miscarriage rates, with highest AMH levels unfavorable effects on live birth rates. A currently widely held opinions is that AMH linearly reflects oocyte quantity and quality, both declining with advancing female age [27, 28]. Here presented AMH observations, however, now suggest otherwise.

Moreover, since miscarriage rates remained the same in all age categories, whether patients produced 1 or 15 embryos, improved outcomes with increasing embryo production (though identical embryo transfer numbers), likely, were independent of embryo quality, as defined by an embryo’s chromosomal integrity. Here presented data, therefore, suggest the existence of yet another embryo quality factor, which is independent of the embryo’s chromosomal status.

The concept of selecting out euploid embryos prior to embryo transfer is the basic principle behind preimplantation genetic screening (PGS) [29]. Here reported findings, therefore, may at least partially explain why, contrary to most predictions, the PGS procedure has so far failed to improve IVF outcomes [30, 31].

The next question to be answered is what drives this previously unknown embryo quality factor, which apparently increases in relative importance with advancing female age? Here presented data suggest that it must be associated with increasing oocyte/embryo production in IVF cycles; yet, since improvements with increasing embryo numbers almost doubled between youngest and oldest age categories, the efficacy of this additional “embryo quality factor” must increase with advancing female age. This observation suggests that AMH may, indeed, be this second, previously unknown “embryo quality factor.”

AMH is, of course, strongly associated with oocyte/embryo production in IVF [27, 28, 32]. At “best” AMH levels, our third model demonstrated extraordinarily high clinical pregnancy rates into even the oldest age categories. Women at ages 41–42 years and above 43 achieved almost unheard of clinical pregnancy rates of 29–30, and 17–18 %, respectively. Neither embryo nor FSH models, however, demonstrated such extraordinary clinical outcomes at advanced age categories.

These extraordinary IVF cycle outcomes, therefore, appear associated with “best” AMH levels, which in this study were defined at ranges of 3.5–8.5 ng/mL in the youngest, and between 4.5 and 7.5 ng/mL in even the oldest age categories.

Yet, in oldest patients this apparently beneficial AMH-associated effect on clinical pregnancy rates was mostly lost to high miscarriage rates. Though live birth rates still remained relatively high until age 42, above age 43, at “best” AMH, they reached only 6–7 %. These rates, though, were still clearly higher than at vey low or very high AMH levels (2–5 %).

## Conclusions

Combined, these observations suggest a “dosage-dependent,” effect of AMH on clinical IVF outcomes: At “best”levels, AMH improves embryo implantation at all ages, leading to peak clinical pregnancy rates. Whether this observation represents an AMH effect on oocytes, embryos or the endometrium remains to be determined. At excessively high levels, AMH, however, to significant degrees appears to increase the risk of pregnancy loss. Miscarriages at highest AMH spiked at all ages to approximately 60 % but reached the incredible rate of 81.8 % in the oldest age category above 43 years.

At “best” levels, AMH, thus meets previously noted requirements for a here newly described “embryo quality factor,” which is quantitatively associated with increasing embryo yields but also increases in efficacy with advancing age. This study, indeed, suggests that AMH, as facilitator and inhibitor, demonstrates increasing utility with advancing female age.

If confirmed by further investigations, here reported effects of AMH on IVF outcomes suggest, especially in older women, at appropriate dosaging a potential therapeutic role in improving clinical outcomes in IVF. Our data, however, also raise the specter of AMH, at higher therapeutic levels, functioning as an abortifaciant.

Somewhat surprisingly, a pharmacological AMH product for human use is currently not available anywhere in the world. This is that more surprising since, at least in animal models, AMH has been demonstrated to demonstrate clinical effects [33].

## Additional file

10.1186/s12967-016-0924-7 Clinical pregnancy and live births between ages <30 and 35 years in reference to embryo numbers.

## Abbreviations

AMH: anti-Müllerian hormone
DHEA: dehydroepiandrosterone
FOR: functional ovarian reserve
FSH: follicle stimulating hormone
hCG: human chorionic gonadotropin
hMG: human menopausal gonadotropin
IVF: in vitro fertilization
LFOR: low functional ovarian reserve
oPOI: occult primary ovarian insufficiency
PGS: preimplantation genetic screening
POA: premature ovarian aging
POI: primary ovarian insufficiency

## References

[1] Kissin DM, Kulkarni A, Kushnir VA, Jamieson DJ (2014). National ART Surveilance System group. Number of embryos transferred after in vitro fertilization and good perinatal outcome. Obstet Gynecol.

[2] Fertility Clinic Success Rate and Certification Act of 1992. HR 4773ENR.

[3] Kushnir VA, Vidali A, Barad DH, Gleicher N (2013). The status of public reporting of clinical outcomes in assisted reproductive technology. Fertil Steril.

[4] Gleicher N, Kushnir VA, Barad DH (2015). Is it time for a paradigm shift in understanding embryo selection?. Reprod Biol Endocrinol.

[5] van Loendersloot L, Repping S, Bossuyt PM, van der Veen F, van Wely M (2014). Prediction models in in vitro fertilization; where are we? A mini review. J Adv Res.

[6] Nelson SM, Lawlor DA (2011). Predicting live birth, preterm delivery, and low birth weight in infants born from in vitro fertilization: a prospective study of 144,018 treatment cycles. PLoS Med.

[7] Bhattacharya S, Maheshwari A, Mollison J (2013). Factors associated with failed treatment: an analysis of 121,744 women embarking on their first IVF cycles. PLoS ONE.

[8] Choi B, Bosch E, Lannon BM, Leveille MC, Wong WH, Leader A, Pellicer A, Penzias AS, Yao MW (2013). Personalized prediction of first-cycle in vitro fertilization success. Fertil Steril.

[9] Van Loendersloot LL, van Wely M, Repping S, Sossuyt PM, van der Veen F (2013). Individualized decision making in IVF: calculating the chances of pregnancy. Hum Reprod.

[10] te Velde ER, Nieboer D, Lintsen AM, Braat DD, Eijkemans MJ, Habbema JD, Vergouwe Y (2014). Comparison of two models predicting IVF success; the effect of time trends on model performance. Hum Reprod.

[11] Smith AD, Tilling K, Lawlor DA, Nelson SM (2015). Extenal validation and calibration of IVF predict: a national prospective cohort study of 130,960 in vitro fertilization cycles. PLoS ONE.

[12] Sunderam S, Kissin DM, Crawford SB, Folger SG, Jamieson DJ, Warner L, Barfield WD (2015). Assisted Reproductive Technology Surveillance—United States, 2012. MMWR Surveill Summ.

[13] Barad DH, Weghofer A, Gleicher N (2007). Age-specific levels for basal follicle-stimulating hormone assessment of ovarian function. Obstet Gynecol.

[14] Barad DH, Weghofer A, Gleicher N (2011). Utility of age-specific serum anti-Müllerian hormone concentrations. Reprod Biomed Online.

[15] Lukaszuk K, Kunicki M, Liss J, Lukaszuk M, Jakiel G (2013). Use of ovarian reserve parameters for predicting live births in women undergoing in vitro fertilization. Eur J Obstet Gynecol Reprod Biol.

[16] Nelson LM (2009). Clinical practice. Primary ovarian insufficiency. N Engl J Med.

[17] Gleicher N, Weghofer A, Barad DH (2011). Defining ovarian reserve to better understand ovarian aging. Reprod Biol Endocrinol.

[18] Gleicher N, Barad DH (2011). Dehydroepiandrosterone (DHEA) supplementation in diminished ovarian reserve (DOR). Reprod Bio Endocrinol.

[19] BenMeir A, Burstein E, BorregoAlvarez A, Chong J, Wong E, Yavorska T, Naranian T, Chi M, Wang Y, Bentov Y, Alexis J, Meriano J, Sung HK, Gasser DL, Moley KH, Hekimi S, Casper RE, Jurisicova A (2015). Coenzyme Q10 restores oocyte mitochondrial function and fertility during reproductive aging. Aging Cell.

[20] Nomura M, Iwase A, Furui K, Kitagawa T, Matsui Y, Yoshikawa M, Kikkawa F (2007). Preferable correlation to blastocyst development and pregnancy rates with a new embryo grading system specific for day 3 embryos. J Assist Reprod Genet.

[21] Gleicher N, Kushnir VA, Barad DH (2014). The danger of ignoring pregnancy and delivery rates in ART. Hum Reprod.

[22] Ethics Committee of the American Society for Reproductive Medicine (2012). Fertility treatment when the prognosis is very poor or futile: a committee opinion. Fertil Steril.

[23] Kushnir VA, Gleicher N (2014). Male factor infertility: prediction models for assisted reproductive technology. Nat Rev Urol.

[24] Seifer DB, Tal R (2015). Personalzed prediction of live birth: are we there yet. Fertil Steril.

[25] Wu Y-G, Barad DH, Kushnir VA, Lazzaroni-Tealdi E, Wang Q, Albertini DF, Gleicher N (2015). Aging-related premature luteinization of granulosa cells is avoided by early oocyte retrieval. J Endocrinol.

[26] Van Helden J, Weiskirchen R (2015). Performance of the two new fully automated anti-Müllerian hormone immunoassays compared with the clinical standard assay. Hum Reprod.

[27] Nelson SM, Fleming R, Gaudoin M, Choi B, Santo-Domingo K, Yao M (2015). Antimüllerian hormone levels and antral follicle count as prognostic indicators in a personalized prediction model of live birth. Fertil Steril.

[28] Tal R, Tal O, Seifer BJ, Seifer DB (2015). Antimüllerian hormone as predictor of implantation and clinical pregnancy after assisted conception: a systematic review and meta-analysis. Fertil Steril.

[29] Wells D (2010). Embryo aneuploidy and the role of morphological and genetic screening. Reprod Biomed Online.

[30] Mastenbroek S, Repping S (2014). Preimplantation genetic screening: back to the future. Hum Reprod.

[31] Gleicher N, Kushnir VA, Barad DH (2014). Preimplantation genetic screening (PGS) still in search of a clinical application: a systematic review. Reprod Biol Endocrinol.

[32] Seifer DB, Tal R (2015). Personalzed prediction of live birth: are we there yet. Fertil Steril.

[33] Maclaughlin DT, Donahoe PK (2010). Müllerian inhibiting substance/anti-Müllerian hormone: a potential therapeutic agent for human ovarian and other cancers. Future Oncol.

