# Supplementary material for: Definition by FSH, AMH and embryo numbers of good-, intermediate- and poor-prognosis patients suggests previously unknown IVF outcome-determining factor associated with AMH
Source: J Transl Med. 2016 Jun 10;14:172. doi: 10.1186/s12967-016-0924-7 (PMC4901433; doi:10.1186/s12967-016-0924-7)
Supplement: Supplementary file 1 — 10.1186/s12967-016-0924-7 Clinical pregnancy and live births between ages <30 and 35 years in reference to embryo numbers. [file 12967_2016_924_MOESM1_ESM.docx]

**Appendix (Supplemental materials)**

Figure S1. Clinical pregnancy and live births between ages <30 and 35 years in reference to embryo numbers

| Clinical pregnancy rates | | | |
| --- | --- | --- | --- |
| Embryos | Ages | | |
|  | <30 | 30-32 | 33-35 |
| 1 | 35% | 32% | 34% |
| 2 | 37% | 34% | 36% |
| 3 | 39% | 35% | 38% |
| 4 | 41% | 37% | 40% |
| 5 | 43% | 39% | 42% |
| 6 | 45% | 41% | 43% |
| 7 | 47% | 43% | 45% |
| 8 | 49% | 45% | 47% |
| 9 | 51% | 47% | 49% |
| 10 | 53% | 49% | 51% |
| 11 | 55% | 51% | 53% |
| 12 | 57% | 53% | 55% |
| 13 | 59% | 55% | 57% |
| 14 | 61% | 57% | 59% |
| 15 | 63% | 59% | 61% |

| Live birth rates | | | |
| --- | --- | --- | --- |
| Embryos | Ages | | |
|  | <30 | 30-32 | 33-35 |
| 1 | 29% | 33% | 28% |
| 2 | 30% | 34% | 29% |
| 3 | 32% | 36% | 31% |
| 4 | 34% | 38% | 32% |
| 5 | 35% | 39% | 34% |
| 6 | 37% | 41% | 36% |
| 7 | 39% | 43% | 37% |
| 8 | 40% | 45% | 39% |
| 9 | 42% | 47% | 41% |
| 10 | 44% | 48% | 42% |
| 11 | 46% | 50% | 44% |
| 12 | 47% | 52% | 46% |
| 13 | 49% | 54% | 48% |
| 14 | 51% | 56% | 50% |
| 15 | 53% | 57% | 51% |

As this figure demonstrates, IVF outcomes were very similar up to age 36 years. Patients under age 36 were, therefore, considered as a single age category.
